# Vitamin D Level and Immune Modulation in Children with Recurrent Wheezing

**DOI:** 10.3390/children11020219

**Published:** 2024-02-08

**Authors:** Gavriela Feketea, Emilia Vassilopoulou, Oana Andreescu, Elena Camelia Berghea, Raluca Maria Pop, Octavia Sabin, Mihnea Zdrenghea, Ioana Corina Bocsan

**Affiliations:** 1 Department of Pharmacology, Toxicology and Clinical Pharmacology, University of Medicine and Pharmacy, 400337 Cluj-Napoca, Romania; g.feketea@karamandaneio.gov.gr (G.F.); raluca.pop@umfcluj.ro (R.M.P.); octavia.sabin@umfcluj.ro (O.S.); bocsan.corina@umfcluj.ro (I.C.B.); 2 Pediatric Allergy Outpatient Clinic, Department of Pediatrics, “Karamandaneio” Children’s Hospital of Patra, 26331 Patras, Greece; 3 Pediatric Unit, Fondazione IRCCS Ca’ Granda Ospedale Maggiore Policlinico, 20122 Milan, Italy; 4 Fundamental, Prophylactic and Clinical Specialties Department, Faculty of Medicine, Transilvania University of Brasov, 500019 Brasov, Romania; oana.andreescu@unitbv.ro; 5 Department of Pediatrics, Carol Davila University of Medicine and Pharmacy, 020021 Bucharest, Romania; bcamelia@gmail.com; 6 Department of Pediatrics, “Marie S. Curie” Emergency Children’s Clinical Hospital, 041451 Bucharest, Romania; 7 Department of Hematology, Iuliu Hatieganu University of Medicine and Pharmacy, 8 Babes Str., 400012 Cluj-Napoca, Romania; 8 Department of Hematology, Ion Chiricuta Oncology Institute, 34-36 Republicii Str., 400015 Cluj-Napoca, Romania

**Keywords:** wheezing, recurrent wheezing, vitamin D, IL-10, IL-31, immune modulation

## Abstract

Introduction and aim: A direct causal relationship between vitamin D (vit D) deficiency and recurrent wheezing has not been proven. The present study investigated the role of vit D in enhancing the risk of asthma or recurrent wheezing by modifying the intensity of the inflammatory process. Material and method: Forty children with wheezing presenting at the emergency service and sixteen healthy control subjects were included in the study. Children with wheezing were either in the first episode (20) or with recurrent wheezing (20). Children with chronic diseases, and other conditions that present with acute wheezing or that might influence the vit D level, were excluded. Blood samples were taken at presentation and 3–6 months later, to evaluate the serum levels of total IgE, vit D, IL-10 and IL-31. Statistical analysis was performed using the SPSS 25 program, with a significance level of *p* < 0.05. Results and conclusion. The vit D level was lower in patients with recurrent wheezing compared with those with a single episode and with the control group, and this increased with time. IL-10 was significantly higher in children with wheezing than in the control group, with the highest values in those with an acute episode of wheezing. IL-31 was higher in children with recurrent wheezing than in those with a first episode only at the initial point, while at the final time point it was lower. Low levels of vit D appear to be detected more frequently in recurrent wheezing than in simple wheezing. Immune modulation, as measured by Th2 status reflected by IL-10 and IL-31 levels, appears to depend on the wheezing phenotype and on the general health status.

## 1. Introduction

Childhood respiratory diseases induced by viruses can be defined in many terms depending on the dominant manifestation, such as acute bronchiolitis, viral lower respiratory tract infection (LRTA), acute viral bronchitis, viral pneumonia, recurrent/transient/nonspecific and/or virally induced wheezing and the exacerbation of asthma [1]. Recurrent wheezing in infants and young children is one of the most common conditions for which parents seek medical attention, and it constitutes a global health problem [2].

In infants and preschool children, wheezing is a frequent clinical symptom of diseases of the respiratory system; it is characterized by a distinctive musical sound in the expiratory phase, and sometimes also in the inspiratory phase, with an increase in respiratory frequency [3]. Wheezing is a multifactorial symptom, and recurrent wheezing in preschool children is largely due to recurrent respiratory tract infection (RTI) [4]. Recurrent wheezing is defined as three or more episodes of this condition in the previous year [5,6] Children who have episodes of wheezing only when they have viral RTIs and have no symptoms between episodes are characterized as having episodic viral wheezing [2,7].

Observational studies have shown that the frequency of asthma increases with latitude [8], as does the prevalence of low levels of vitamin D (vit D) in childhood [9]. Epidemiological studies have indicated that low serum levels of vit D are associated with a higher risk of respiratory infections in children [10]. Experimental and clinical studies document positive effects of vit D on the immune system, and on the response to the treatment of asthma [11]. Several studies have reported that vit D deficiency may lead to an increased frequency of wheezing episodes [12,13], and the need for more medication. Vit D sufficiency, insufficiency and deficiency are defined as serum levels of 25(OH)D of >30 ng/mL, 20–30 ng/mL and <20 ng/mL, respectively, [>75 nmol/L, 50–75 nmol/L and <50 nmol/L, respectively] [14]. Supplementation with vit D may protect children against viral infections and decrease the number and/or severity of recurrent wheezing episodes [15,16]. Studies in the Northeastern US have shown that increased maternal vit D intake during pregnancy, from either dietary sources or supplements, may lower the risk of wheezing in early childhood [17,18]. However, a recent updated Cochrane review found no evidence to support the administration of vit D supplementation to reduce the risk of exacerbations in mild and moderate asthma [19]. Most reported associations between vit D levels and infectious disease outcomes, including respiratory tract infections, were not statistically significant [20].

The immune modulation observed during infection and inflammation takes a variety of forms. Interleukin 10 (IL-10), a cytokine with anti-inflammatory properties closely related to T regulatory (T reg) cells, plays an important role in Th1–Th2 balance, limiting the immune response to pathogens, and in this manner preventing local damage [21,22]. IL-31 is a relative novel cytokine produced by Th2 cells involved in the pathogenesis of bronchial inflammation [23]. It seems that IL-31 might have a dual effect on Th2-type inflammation depending on disease stage [24]. An increased IL-31 could lead to a Th2-dominant inflammation in the early stage, but with late anti-inflammatory action in asthma [25] It is also a potential biomarker for the phenotypic classification of viral bronchiolitis, depending on the type of virus [26]. 

## 2. Hypothesis and Aims of the Study

To date, a direct causal relationship between vit D deficiency and recurrent wheezing has not been proven. Most relevant studies report that deficiency or insufficient levels of vit D predispose to wheezing episodes and exacerbations of asthma [27]. Infections may result as a consequence of low vit D levels, due to a decrease in its synthesis, which could be a result of the reduced exposure to sunlight of children who spend more time indoors [28]. It can also be speculated that the low level of vit D in these children is due to excessive consumption of vit D in the immune processes that occur during asthma exacerbations or wheezing episodes, but this remains to be proven. The present study aimed to analyze the level of vit D and some Th2 inflammatory parameters in children with recurrent wheezing. The main purpose is an exploration of whether vit D deficiency may influence the severity of the inflammatory process and may correlate with wheezing severity in two groups of children with different wheezing phenotypes. After a period of 4–6 months without further infections, vit D and inflammatory parameters levels should return to normal, or there should be no significant difference between the studied groups. 

## 3. Methods

### 3.1. Study Population

This was a cross-sectional, analytical study of children who presented consecutively at the emergency service of the regional Hospital of Amaliada, Greece, between September 2019 and February 2020, with wheezing as the main complaint. The study evaluated, over time, vit D levels in children with multiple episodes of wheezing compared with those who had a single episode and with healthy children. The children with wheezing were assigned to two groups: group 1 consisted of 20 patients who presented with a first episode of wheezing, and group 2 of 20 patients with recurrent wheezing, defined as more than 3 episodes of wheezing within the last 3 months. A control group was recruited of 16 healthy children who attended the Department of Pediatrics for regular check-ups and mandatory vaccinations in the same time period (during the winter), and had no history of chronic disease, lower respiratory tract disease or wheezing. The entire study population lived in the same region and reported no other episode of wheezing between the initial and final time point, and no differences were recorded in religious and alimentary practices affecting dietary vit D intake. The exclusion criteria were chronic diseases, musculoskeletal and congenital diseases, genetic syndromes, malabsorption disorders and prematurity; children with bronchiectasis, gastroesophageal reflux, swallowing disorders, foreign body aspiration, primary and secondary immunodeficiencies, malignancy and neurological diseases were also excluded. In addition, children with a severity of wheezing that required hospitalization were not included in this study. In order to reduce the influence of other factors that could be associated with wheezing [29], children were also excluded that had taken antibiotics for some reason in the first 2 months of life, and vit D supplements in the last 3 months, those with obesity, i.e., body mass index (BMI) >28, and those whose parents smoke or who had clinical signs of allergy. 

The study was approved by the Scientific and Ethics Council of the Amaliada Hospital Unit, General Hospital of Ilia, Greece, and by the Ethics Commission of the “Iuliu Hațieganu” University of Medicine and Pharmacy, Cluj Napoca, Romania. Before recruitment, the parents provided their written informed consent for participation of their children in the study. 

### 3.2. Study Design and Data Collection

The data recorded for each child included age, sex, type of birth, type of nutrition in the first 3 months of life (breastfeeding, artificial milk formula or mixed feeding) and clinical data related to the current acute illness: duration and amplitude of fever, and details of signs and symptoms characteristic of an RTI (i.e., cough, chest pain, shortness of breath, myalgia, headache, sore throat, rhinorrhea, diarrhea, nausea, vomiting). The respiratory rate, heart rate and oxygen saturation at presentation were also recorded.

Venous blood samples were collected from each child with wheezing at presentation (basal) and at 3–6 months after the wheezing episode (final evaluation) and the following parameters were determined: blood cell count, erythrocyte sedimentation rate (ESR), serum levels of C-reactive protein (CRP), calcium, alkaline phosphatase, vit D [25-hydroxycholecalciferol, 25(OH)D], IL-10 and IL-31, total immunoglobulin E (IgE) and liver function tests (SGOT, SGPT). Blood samples were taken from the children in the control group at recruitment and after 3–6 months, at the time of routine testing. The biochemical measurements and hemogram were performed using a Unicel DxH 600 Coulter Cellular Analysis System by Beckman Coulter, Krefeld, Germany, for the blood count and a Siemens The Dimension^®^ RxL Max^®^ Integrated Chemistry System, Munich, Germany, for the other determinations. A part of each sample after centrifugation at 3500 rpm for 15 min was stored at −80 °C, until the determination of the serum levels of 25(OH)D, IL-10 and IL-31 and total IgE.

Total IgE was determined by the electrochemiluminescence technique, using a Cobas e-411 analyzer. Vit D (25(OH)D), IL-31 and Il-10 were determined by ELISA technique. The following determination kits were used: 25(OH)D Total ELISA Kit (DIAsource ImmunoAssays, Louvain-la-Neuve–Belgium), Human Quantikinine IL-10 (R&D system) and Human Il-31 Duoset ELISA (R&D System, Minneapolis, MN, USA). The samples and standard dilutions were assayed according to the manufacturer’s instructions. 

### 3.3. Statistical Analysis

Statistical analysis was conducted using the SPSS 25 program. The distribution of continuous variables was checked using the Kolmogorov–Smirnov normality test and they were characterized as mean and standard deviation (±SD) or median and 25–75th percentiles. Nominal variables were expressed as number and percentage. Quantitative variables were compared using the non-parametric Mann–Whitney and Wilcoxon tests. Nominal variables were compared using the Chi-square test or Fisher’s Test. The level of statistical significance was set at *p* < 0.05.

## 4. Results

The demographic data of the children included in the study are presented in Table 1. Eleven girls (55%) and nine boys (45%), with a median age of 3.37 (1.5–5) years presented with the complaint of wheezing with no previous episode (group 1). Seven girls (35%) and thirteen boys (65%) with a median age of 3.12 (1.5–5) years, who presented with wheezing, had a history of more than three recurrent episodes of wheezing in the last 3 months (group 2). In the healthy control group (group 3), eight were girls (50%) and eight were boys (50%), with a median age of 10.59 (9–14) years. No gender distribution difference was found between the groups (*p* > 0.05) (Table 1). The control group had a higher median age than the patient groups. 

The laboratory data of the children in the study are presented in Table 2. 

**Figure 1:**
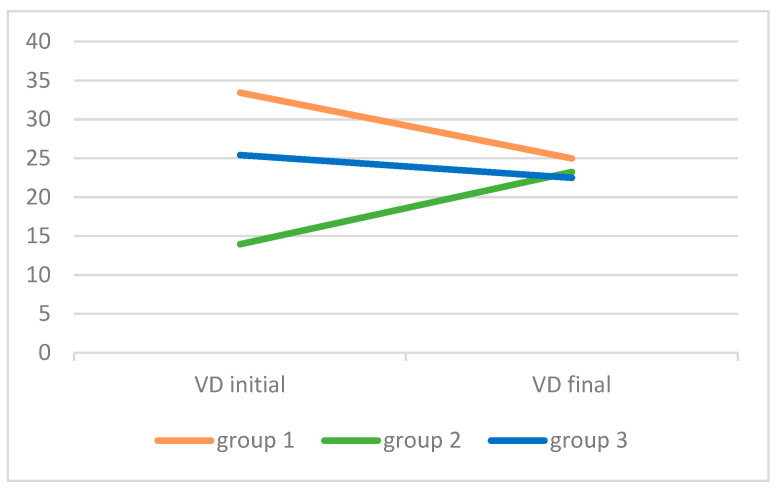
Level of Vitamin D [25(OH)D, VD] in the three study groups of children at the beginning and the end of the study (3–6 months after the wheezing episode). Group 1: single episode of wheezing (N = 20); group 2: recurrent wheezing (N = 20); group 3: control group (N = 16). All values are median.

Between the initial and the final measurements, the median level of vit D decreased in group 1 (single episode of wheezing) and in the control group, while in children with recurrent wheezing, it was very low initially, but increased, reaching a level similar to the other two groups by the final measurement 3–6 months later (Table 2, Figure 1). 

Between the two groups of patients with wheezing, IL-31 was higher in group 2, the children with recurrent wheezing, at the initial time point, but lower at the final time point. 

IL-10 was lower in children with recurrent wheezing than in group 1, both at initial presentation and at the end of the study.

At the initial evaluation, MPV, ESR and levels of CRP, IL-10 and vit D showed significant differences between the studied groups, in as shown in Table 3. Also, IL-31 was lower in the control group vs. for children with wheezing, but the differences were not statistically significant. Vitamin D level was positively correlated with IL-31 median values in children with acute wheezing (R = 0.499, *p* = 0.025), but not in those with recurrent wheezing (*p* > 0.05).

However, after 3–6 months, no statistically significant difference between the three groups was detected for the aforementioned parameters, as shown in Table 4. Analyzing the evolution of the cytokines, a discordant tendency was noted for IL-31, which increased in children with wheezing and in controls, while in those with recurrent wheezing it decreased, but these differences were not significant between groups or in comparison with basal values. 

## 5. Discussion

Epidemiological and observational studies have reported a consistent association between vit D deficiency and viral respiratory infections under certain conditions, and interventional studies on vit D supplementation and/or vit D status have produced mixed and sometimes conflicting findings [30]. Wheezing in children is most often caused by acute RTI [31], and recurrent wheezing in children aged under 5 years is a heterogeneous condition, usually associated with recurrent upper RTIs. The wheezing phenotypes proposed by the Task Force of the European Respiratory Society (ERS) in 2008 differ depending on the criteria used for classification, namely episodic viral or multiple-trigger wheezing, depending on symptoms, and transient, persistent and late-onset wheezing, according to time-trend classification [2]. This approach allows individual therapeutic decisions to be made, based on the temporal pattern of symptoms [32]. In daily clinical practice, however, many infants and young children present with wheezing during viral infections, and thus a classification into one of these phenotypes is unrealistic [7]. The identification of those infants with wheezing who are at risk of future recurrence and/or severe progression could help pediatricians to improve their therapeutic decisions.

Deng and colleagues characterized factors that are probably associated with wheezing and asthma in preschool children [29], which include the administration of antibiotics in the first month of life, a personal and/or family history of allergy or atopy, and obesity. In our study, the children in whom the above factors were present were excluded. Regarding vit D, the same authors found significant differences in levels in children who did not present wheezing and those who received vit D supplements [29].

Demirel and colleagues detected lower levels of vit D in children with recurrent wheezing compared with a control group [13]. Other studies have reported that low levels of vit D may be a risk factor for recurrent wheezing [33,34]. In the present study, the median vit D level at baseline was the lowest in the group of children with recurrent wheezing. Also, the median vit D level was higher in the children with a single episode of wheezing than in the control group. This could be explained by the fact that the control group consisted of older children, and in winter the serum level of vit D is reported to be lower in older children [35], as confirmed by the authors in previous studies [36,37]. In our study, significantly lower levels of vit D were found in children with recurrent wheezing than in either of those with a single episode, or in the control group, only at baseline evaluation. At the final time point, the three groups had similar serum vit D levels. The trend of vit D was different in the studied groups. The children with a single episode of wheezing, and the control group, showed a descending trend of vit D, possibly dependent on the season. A similar descending trend of vit D level was also observed by Forno and colleagues, even in their vit D supplementation group [38]. The study children with recurrent wheezing showed an increasing trend in vit D after the wheezing stopped, even in those without vit D supplementation. We could speculate that after repeated episodes of wheezing, the vit D level returned in a relatively short time to levels similar to those observed in the children with a single episode of wheezing, and then may have followed a similar descending trend, corresponding to the seasonal variation, reaching approximately the same values at the end of the study. This explanation could support the theory that during episodes of wheezing and during infections, the level of vit D decreases due to imbalance between the supply (constant or even low, due to isolation at home) and the increased consumption in the immune processes activated during infection. Unfortunately, we did not predict this possibility, and failed to include in the study an intermediate blood test, 1–2 weeks after the onset of the acute episode.

Relevant studies have suggested that IL-31 may be involved in supporting allergic inflammation and is associated with a specific airway epithelial cell response that may characterize allergic asthma [39,40,41]. In one study, children with a history of more than three episodes of wheezing before the age of 2 years were later diagnosed with asthma much sooner [42]. The level of IL-31 could therefore be expected to be higher in children with recurrent wheezing. In our study, however, the level of IL-31 was higher in children with recurrent wheezing than in those with a first episode only at the initial point, while at the final time point, it was lower. A plausible explanation would be that the children in our study were older than those in the cited study and were in full health at the final time point, 3–6 months after the episode, with no further infections.

The evolution of IL-31 was discordant, in some groups having an ascending trend and in others a descending one. IL-31 seems to have a dual role in asthma, a pro-inflammatory one in the early stage and anti-inflammatory action in the late one. IL 31 is a cytokine that increases in eosinophilic inflammation, i.e., for RV bronchiolitis [24]. In the present study, children had wheezing or recurrent wheezing but not asthma, so we might speculate that IL-31 may play a role when allergic, eosinophilic inflammation is already settled, without having a role in preventing its development. Another explanation for this non-significance can be based on the fact that cases with clinical signs of allergy were excluded, so children with a possible minimal eosinophilic inflammation were not included in the present study. The present study showed that IL-31 does not influence the evolution of pre-existing asthma conditions in the absence of allergic inflammation.

IL-10 is a cytokine with regulatory properties in the immune response, produced by dendritic cells (DCs) and macrophages. The early production of IL-10 by antigen-presenting cells appears to limit excessive inflammation, and thus possible tissue damage [43]. During acute infections, proinflammatory signals are generated by DCs to recognize pathogen patterns. In this pro-inflammatory context, dendritic cells can promote antiviral T-cell responses responsible for pathogen and infection clearance. The activation of these DCs and of natural killer (NK) cells also results in the production of the IL-10 to balance the inflammatory process [44]. Loebbermann and colleagues demonstrated that respiratory syncytial virus (RSV) infection induced IL-10 production by CD4(+) and CD8(+) T cells in mice [45]. In the present study, the mean IL-10 levels were higher in the initial measurements at the onset of wheezing than at the final phase 3–6 months later, in both groups with wheezing, consistent with the antiviral activity of this cytokine. Bont and colleagues showed that IL-10 levels, measured in the convalescent phase of RSV bronchiolitis in infants 3–4 weeks after the hospitalization, were significantly higher in children who developed recurrent wheezing during the following year than in those without recurrence [46]. Conversely, in our study, IL-10 levels were lower in children with recurrent wheezing, but older age, viral etiology other than RSV and milder severity, not requiring hospitalization, are possible reasons for these differences.

This study highlighted the involvement of vit D in immune modulation and raised the hypothesis of a possible indirect relationship between vit D level and Th2-specific immune response in children with recurrent wheezing who are at possible risk of developing asthma. Vit D positively correlated with IL-31 in the acute phase of wheezing, but not for the long term. It seems that the immune response was normal outside the viral infections, and no minimal variation in IL-10 and IL-31 was noticed in the end of the study. This could be explained by the fact that IL-10 and IL-31 could play a role in augmenting or limiting the inflammation when it is already settled at the beginning of the acute episode, having no role in preventing the development of allergic inflammation long-term. 

This study has several limitations. Firstly, the study groups of children were not large. Due to the COVID-19 pandemic, the process of inclusion and monitoring of patients had to be stopped, and many children already recruited were excluded because of the impossibility of final monitoring at the 6-month time-point. In addition, the children in the control group, because of the small numbers of younger children attending the hospital for routine visits, were much older than those with wheezing. Another limitation was the lack of specific virus determination. Only children with viral etiology of wheezing were recruited, excluding those with bacterial infection, but the technical possibility of determination of the type of virus was missing. 

## 6. Conclusions

Recurrent wheezing in children is a common reason for specialist referral. The identification of those infants and young children with wheezing who are at increased risk of recurrence and possible development of asthma could help specialists to improve their therapeutic decisions. Low levels of vit D appear to be detected more frequently in recurrent wheezing, and therefore the determination of serum 25(OH)D may be a useful biomarker in infants and children presenting with wheezing. The routine monitoring of serum 25(OH)D should be considered in children with recurrent wheezing, and only in the case of deficiency should this be corrected. It remains unclear whether the low vit D level in children with wheezing predisposes to relapse, or that the recurrent episodes of wheezing result in lowering the level of vit D. Immune Th2 status reflected by IL-10 and IL-31 levels appears to depend on the wheezing phenotype, on the phase of the disease and on the general health status, but larger studies are needed to further explore their role in children with recurrent wheezing. 

## Figures and Tables

**Table 1:** Demographic data of children with wheezing (group 1), recurrent wheezing (group 2) and healthy children (group 3—control).

	Group 1—Wheezing N = 20	Group 2—Recurrent Wheezing N = 20	Group 3—Control N = 16	*p*
Sex No (%)				
Male	9 (45%)	13 (65%)	8 (50%)	
Female	11 (55%)	7 (35%)	8 (50%)	*p* > 0.05
Age (Median/percentiles)	3.37 (1.5–5)	3.12 (1.5–5)	10.59 (9–14)	*p* < 0.001

Years of age are presented as median and (25th–75th) percentiles.

**Table 2:** Laboratory data of children with wheezing (group 1), recurrent wheezing (group 2) and healthy children (group 3—control) at presentation (initial) and 3–6 months after the wheezing episode (final).

	Group 1—Wheezing N = 20			Group 2—Recurrent Wheezing N = 20			Group 3—Control N = 16		
	Initial	Final	*p*	Initial	Final	*p*	Initial	Final	*p*
Total leucocyte count (/mm^3^)	8150 (6600–14,150)	6700 (5725–7825)	0.041	7800 (4700–9400)	6500 (5800–7700)	0.221	6200 (5175–8650)	6400 (5400–8050)	0.67
Neutrophils (/mm^3^)	4650 (2800–6700)	3150 (2650–4125)	0.027	4550 (2850–5725)	3700 (2550–4450)	0.114	3400 (2725–4250)	3700 (2700–4375)	0.08
Lymphocytes (/mm^3^)	2600 (1725–3925)	2450 (1550–3187)	0.65	2350 (1725–2900)	2200 (1700–2950)	0.559	2100 (1800–3350)	1900 (1600–2300)	0.59
Monocytes (/mm^3^)	650 (500–1325)	650 (500–950)	0.284	600 (600–700)	500 (500–650)	0.105	500 (425–600)	600 (500–900)	0.94
PLT (×10^9^)	275.5 (207.25–347.50)	293 (271–337)	0.905	277.5 (246.50–340.00)	255 (214–290)	0.118	314.0 (248.5–370.5)	290 (224–350)	0.52
MPV (fL)	7.9 (7.32–8.3)	8.2 (7.35–8.4)	0.184	8.85 (8.05–9.67)	8.8 (7.75–9.7)	0.48	8.45 (8.10–9.07)	8.2 (7.8–8.95)	0.45
CRP (mg/L)	1.1 (0.27–3.47)	0.25 (0.1–0.4)	0.005	1.8 (0.72–2.10	0.4 (0.15–0.5)	<0.001	0.10 (0.10–0.20)	0.35 (0.2–0.47)	0.83
ESR (mm/h)	26 (10–37.75)	12 (9–14)	0.006	25 (19.25–28.00)	12 (10.5–19.5)	0.004	9 (6.25–12)	10 (7–11.5)	0.79

PLT: platelets; MPV: mean platelet volume; CRP; C-reactive protein; ESR: erythrocyte sedimentation rate; IL-10: interleukin 10; IL-31: interleukin 31. All values are presented as median and (25–75th) percentiles.

**Table 3:** Levels of vitamin D, MPV, ESR, CRP and IL-10 in the three study groups of children on presentation.

	Group 1 (Wheezing, N = 20)	Group 2 (Recurrent Wheezing, N = 20)	Group 3 (Control, N = 16)	*p*
MPV (fL)	7.9 (7.32–8.3)	8.85 (8.05–9.67)	8.45 (8.1–9.07)	0.033
ESR (mm/h)	26 (10–38)	25 (19–28)	9 (6–12)	0.001
CRP (mg/L)	1.1 (0.27–3.47)	1.8 (0.72–2.1)	0.1 (0.1–0.2)	<0.001
IL10 (pg/mL)	22.03 (16.17–39.54)	17.81 (11.06–37.79)	5.35 (4.32–8.19)	<0.001
IL-31 (pg/mL)	7490.18 (2307.49–11,853.75)	7780.54 (2581.32–11,119.61)	5844.84 (3235.20–10,813.91)	>0.05
vit D level (ng/mL)	33.44 (23.1–46.38)	13.94 (11.92–23.27)	25.42 (21.22–32.95)	<0.001

MPV: mean platelet volume; CRP; C-reactive protein; ESR: erythrocyte sedimentation rate; IL-10: interleukin 10; vit D: 25(OH)D. All values are presented as median and (25th–75th) percentiles.

**Table 4:** Levels of vitamin D, MPV, ESR, CRP and IL-10 in the three study groups of children at the end of the study (3–6 months after the wheezing episode).

	Group 1 (Wheezing, N = 20)	Group 2 (Recurrent Wheezing, N = 20)	Group 3 (Control, N = 16)	*p*
MPV (fL)	8.2 (7.35–8.4)	8.8 (7.75–9.7)	8.2 (7.8–8.95)	>0.05
ESR (mm/h)	12 (9–14)	12 (10–19)	10 (7–11)	>0.05
CRP (mg/L)	0.25 (0.1–0.4)	0.4 (0.15–0.5)	0.35 (0.2–0.47)	>0.05
IL10 (pg/mL)	22.97 (17.86–41.72)	14.93 (12.67–42.85)	15.75 (12.4–30.12)	>0.05
IL-31 (pg/mL)	8842.82 (2544.73–11,830.15)	7112.48 (2612–9776.42)	9779.97 (3333.17–11,019.28)	>0.05
vit D level (ng/mL)	24.98 (21.95–35.96)	23.25 (17.34–29.34)	24.98 (18.54–31.12)	>0.05

MPV: mean platelet volume; CRP; C-reactive protein; ESR: erythrocyte sedimentation rate; IL-10: interleukin 10; vit D: 25(OH)D. All values are presented as median and (25–75th) percentiles.

## Data Availability

The data that support the findings of this study are available on request from the corresponding author. The data are not publicly available due to privacy and ethical restrictions.
